# Neonatal Isoflurane Does Not Affect Sleep Architecture and Minimally Alters Neuronal Beta Oscillations in Adolescent Rats

**DOI:** 10.3389/fnbeh.2021.703859

**Published:** 2021-11-01

**Authors:** Francesca M. Manzella, Bethany F. Gulvezan, Stefan Maksimovic, Nemanja Useinovic, Yogendra H. Raol, Srdjan M. Joksimovic, Vesna Jevtovic-Todorovic, Slobodan M. Todorovic

**Affiliations:** ^1^Department of Anesthesiology, University of Colorado Anschutz Medical Campus, Aurora, CO, United States; ^2^Neuroscience Graduate Program, University of Colorado Anschutz Medical Campus, Aurora, CO, United States; ^3^National Institute of Neurological Disorders and Stroke, National Institutes of Health, Rockville, MD, United States; ^4^Department of Neurology, Perelman School of Medicine, University of Pennsylvania, Philadelphia, PA, United States; ^5^Division of Child Neurology, CHOP Research Institute, Children’s Hospital of Philadelphia, Philadelphia, PA, United States

**Keywords:** anesthesia, neurotoxicity, sleep, EEG, beta oscillations, neurotoxicity

## Abstract

General anesthetics are neurotoxic to the developing rodent and primate brains leading to neurocognitive and socio-affective impairment later in life. In addition, sleep patterns are important predictors of cognitive outcomes. Yet, little is known about how anesthetics affect sleep-wake behaviors and their corresponding oscillations. Here we examine how neonatal general anesthesia affects sleep and wake behavior and associated neuronal oscillations. We exposed male and female rat pups to either 6 h of continuous isoflurane or sham anesthesia (compressed air) at the peak of their brain development (postnatal day 7). One cohort of animals was used to examine neurotoxic insult 2 h post-anesthesia exposure. At weaning age, a second cohort of rats was implanted with cortical electroencephalogram electrodes and allowed to recover. During adolescence, we measured sleep architecture (divided into wake, non-rapid eye movement, and rapid eye movement sleep) and electroencephalogram power spectra over a 24 h period. We found that exposure to neonatal isoflurane caused extensive neurotoxicity but did not disrupt sleep architecture in adolescent rats. However, these animals had a small but significant reduction in beta oscillations, specifically in the 12–20 Hz beta 1 range, associated with wake behavior. Furthermore, beta oscillations play a critical role in cortical development, cognitive processing, and homeostatic sleep drive. We speculate that dysregulation of beta oscillations may be implicated in cognitive and socio-affective outcomes associated with neonatal anesthesia.

## Introduction

The use of traditional anesthetics is necessary for life saving interventions in infants and young children. These agents are typically *N*-methyl-D-aspartate (NMDA) receptor antagonists, such as ketamine, or γ-aminobutyric acid type A (GABA_A_) receptor agonists such as isoflurane. Although used less frequently in developed nations, isoflurane is one of the most commonly used anesthetics in the world, especially in the developing countries. Because of affordability, industry reports show that demand for isoflurane is increasing globally. However, it is well established that exposure to isoflurane and other traditional anesthetics is neurotoxic to the developing rodent and primate brain (Jevtovic-Todorovic et al., 2003; Noguchi et al., 2017; Schenning et al., 2017). In addition to causing short-term neurotoxic insult in animal models, anesthetics are also associated with long-term cognitive deficits (Paule et al., 2011; Brambrink et al., 2012; Creeley et al., 2013; Maloney et al., 2019). Newest meta-analysis data suggest that infants and young children exposed to anesthetics during critical stages of their brain development may be at increased risk for developing neurocognitive and socio-affective impairments (Ing et al., 2021). However, little is known about the effects of neonatal general anesthesia on other behavioral and functional outcomes, such as sleep-wake behavior and associated electroencephalogram (EEG) oscillations, which in turn can affect cognition. Yet, exposure to neonatal general anesthesia causes neurotoxicity in brain regions that regulate awareness and sleep, such as neocortex, thalamus, and hypothalamus (Jevtovic-Todorovic et al., 2003). In addition to neurotoxic insult, these regions are also potentially susceptible to anesthetic induced changes in plasticity and excitability. For example, our previous studies have shown that neonatal exposure to general anesthetics results in changes in plasticity of synaptic and intrinsic ionic currents in the nucleus reticularis (nRT) and the ventrobasal (VB) nucleus of the thalamus in neonatal and juvenile rats (DiGruccio et al., 2015; Joksovic et al., 2015; Woodward et al., 2019). This is important since both VB and nRT nuclei of the thalamus play a major role in the corticothalamic tract regulating slow wave sleep and associated oscillations, such as delta waves (Steriade, 2006).

Various brain oscillations during sleep are associated with sleep quality and neurocognitive performance. For example, slower frequencies in the range of delta oscillations during non-rapid eye movement (NREM) sleep play a role in experience plasticity and memory consolidation (Binder et al., 2014; Wilhelm et al., 2014). They also play a role in regulating the homeostatic sleep drive (Vyazovskiy et al., 2007). Similarly, intermediate frequencies in the range of beta activity during quiet wake couples with slow waves to regulate homeostatic sleep (Grønli et al., 2016). It is well known that sleep deprivation and poor sleep quality is associated with memory impairment, especially in children and adolescents (Carskadon, 2011; Ward et al., 2017). Thus, exposure to anesthetics at a young age can potentially affect sleep behavior and sleep quality, which may contribute to cognitive impairments.

The function of neuronal oscillations are not limited to regulating sleep. For example, in addition to their role in sleep homeostasis, beta oscillations are also associated with attention regulation, decision making, and learning during wake states (Zaepffel et al., 2013; Schutte et al., 2017; Park et al., 2018). Consequently, treatment with anesthetics during the neonatal period can disrupt oscillatory patterns during wake, especially during cognitive tasks. Dysregulation of neuronal oscillations during sleep and awake may further perpetuate the risk of neurocognitive impairment associated with exposure to neonatal anesthetics.

Since our recent studies showed that general anesthesia during brain development induces lasting plasticity of ion channels in the rat thalamus, we hypothesized that neonatal isoflurane exposure may have an effect on sleep and wake oscillations as well as sleep and wake behaviors in female and male adolescent rats. We used cortical EEG analysis to characterize neuronal oscillations during normal sleep-wake cycles. We found that exposure to 6 h of 1.5% isoflurane at postnatal day (P) 7 causes extensive neurotoxicity but does not alter sleep architecture. However, we found that neonatal anesthesia exposure did cause small but significant alterations in beta oscillations during the wake stage. These results suggest that neonatal exposure to isoflurane alters oscillatory patterns during wake behavior, which may in turn affect cognitive outcomes.

## Materials and Methods

### Anesthesia Exposure

**FIGURE 1:**
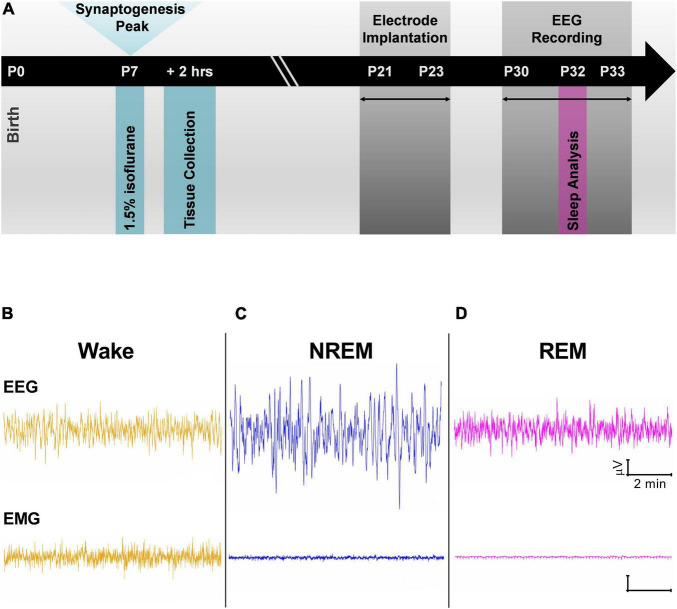
**(A)** Schematic representation of experimental timeline. Exposure to 1.5% Isoflurane for 6 h occurred at P7 during peak synaptogenesis. One cohort of animals was used to quantify neuroapoptosis 2 h after anesthesia exposure. A second cohort of animals was raised with their mothers until weaning age before being implanted with electroencephalogram (EEG) electrodes. Adolescent rats were given at least a week to recover from surgeries, and at P30 EEG recordings began. Data from P32 and P33 were used in all analyses. **(B–D)** Cortical EEG and electromyography (EMG) representative traces were used alongside time-locked videos to score sleep. **(B)** Traces during wake have mixed frequency/low amplitude EEG and represent an animal that was freely and actively moving as noted by its high frequency EMG. **(C)** Representative traces during non-rapid eye movement (NREM) show low frequency cortical oscillations and minimal movement in EMG. **(D)** Cortical oscillations during rapid-eye movement sleep (REM) are similar to those of Wake stage but with flat line in the EMG.

All experiments were approved by the Institutional Animal Care and Use Committee at the University of Colorado Anschutz Medical Campus and adhered to the NIH Guide for the Care and Use of Laboratory animals. Anesthesia exposure experiments were conducted in Sprague-Dawley (Envigo, United States) rat pups of both sexes. Pups were housed with their mothers until initiation of experiment. Animals were maintained on a 14:10 light-dark cycle and had access to food and water *ad libitum*. At P7, rat pups were removed from their home cages and randomly assigned to receive exposure to either the anesthesia condition or the sham condition. This time period corresponds to the peak of synaptogenesis when animals are most vulnerable to the effects of anesthesia (Jevtovic-Todorovic et al., 2003). Those in the anesthesia condition were exposed to 6 h of a volatile anesthetic, 1.5% isoflurane, which was delivered in compressed air containing 22% O_2_ (Iso group). Previous work has found that this dose of isoflurane is sufficient for inducing apoptosis in a rodent model (Johnson et al., 2008). Pups in the sham condition were only delivered compressed air (Sham group). Gas levels (isoflurane, oxygen, and carbon dioxide) in the Iso and Sham chambers were measured with a Datex Capnomac Ultima monitoring system. A heating blanket adjusted to 35°C was used to keep the pups warm during exposure period. Following the 6-h exposure, Iso and Sham pups were allowed to recover and returned to their mothers (Figure 1A, P7 time point). Two cohorts of pups were used, one for describing acute neuroapoptosis, and a second for EEG experiments.

### Assessment of Developmental Neuroapoptosis

After exposure to either anesthesia or room air, all rat pups had a resting period of 2 h prior to transcardial perfusion (Figure 1A, 2 h timepoint). Animals were deeply anesthetized using isoflurane and then perfused with 4% paraformaldehyde (PFA) in phosphate buffered saline (0.1 M), pH 7.4 for immunohistochemistry studies. Brains were kept in 4% PFA for 24 h, embedded in 3% agarose, and sectioned in the coronal plane in 50-μm-thick vibratome sections. Because the ventrobasal thalamus plays a critical role in sleep oscillations, we took ventral posterior medial and lateral thalamus sections for activated caspase 3 (AC3) staining, a marker of neuronal apoptosis. To establish consistent anesthesia induced neurotoxicity, we also took sections of dorsal subiculum, which we have previously shown is vulnerable to neonatal anesthesia (Jevtovic-Todorovic et al., 2003; Atluri et al., 2018). Sections were washed in 0.01 M phosphate-buffered saline (PBS) and quenched for 10 min in a Bloxall^®^ solution (Endogenous Blocking Solution, Peroxidase and Alkaline Phosphatase SP-6000-100, Vector Labs). Subsequently, sections underwent incubation for 1 h in blocking solution (5% goat serum/0.1% Tween-20 in PBS). Slices were then incubated overnight with a primary anti-active caspase-3 antibody [Cleaved Caspase-3 (Asp175) (5A1E) Rabbit mAb (Biotinylated), Cell Signaling Technology] diluted 1:100 in blocking solution (1% goat serum/0.1% tween-20 in PBS) at 4°C. After incubation with biotinylated antibody, sections were reacted in the dark with a Vectastain ABC reagent [Vectastain ABC HRP Elite Kit (Peroxidase, Standard), Vector Labs] for 1 h. The reaction complexes were developed by incubating in DAB (3,3-diaminobenzidine) substrate [ImmPACT DAB Peroxidase (HRP) Substrate, Vector Labs] following the manufacturer’s instructions.

### Quantitative Immunohistochemistry

In order to determine the number and density of AC3 stained neurons in a given brain region, the sections were scanned at 20× magnification using a Nikon Eclipse 800 microscope with the camera. We examined aforementioned regions of the brain using a postnatal rat pup brain atlas (Paxinos and Watson, 2004): hippocampal CA1-Subiculum junction (–5.80 mm from bregma); VB complex (–2.64 mm from bregma). AC3 positive neurons were counted manually using a 500 μm^2^ grid in the region of interest and multiplied by two to express the density of AC3 neurons per 1 mm^2^. The counting was done by an investigator blinded to the experimental conditions using NIS-Elements BR-5.11.02 software, Tokyo, Japan.

### Electrode Implantation and Electroencephalogram Acquisition

To study the effects of neonatal anesthesia on sleep, Iso, and Sham animals underwent stereotaxic surgeries between P21 and P23 (Figure 1A, P21 time point). Anesthesia was induced at 3% isoflurane via inhalation and maintained between 0.5 and 2%. Lidocaine (1%) was applied at the incision site as a local anesthetic. Because both groups were past the critical period for anesthesia induced neurotoxicity, there was no concern in exposing the Sham group briefly to isoflurane for surgery. A screw electrode was implanted with stereotaxic coordinates in the range of barrel cortex: AP –0.8 to –2.4 mm, ML 3.0 mm. Two screw electrodes were implanted just caudal to lambda: one left of the midline, which served as the reference electrode and one right of the midline, which served as the ground electrode. An insulated silver wire hook was inserted in the nuchal muscle to measure electromyography (EMG). For use in another experiment, animals were also implanted with a depth electrode either in the subiculum (AP –4.2 mm, ML 2.0 mm, and DV 2.5 mm) or in the VB (–3.0 mm, ML 2.8 mm, and DV 5.4 mm). Dental acrylic was used to fix the electrodes to the skull, forming the EEG headpiece. Animals were treated post-operatively with Banamine (2.5 mg/kg subcutaneously) for analgesia every 24 h for 48 h.

Animals were housed individually and given at least a week to recover from surgery and to adjust to the headpiece. EEG recording and acquisition took place between P30 and 33 (Figure 1A, P30 time point). EEG signals were recorded using the Pinnacle system (Pinnacle Technology, Inc., Lawrence, KS, United States) alongside synchronized, time-locked video. We acquired the EEG signals using a 1–500 Hz bandpass filter, which were digitized at 2,000 Hz and stored on a hard disk for offline analysis.

### Sleep Behavior and Analysis

Electroencephalogram and EMG for sleep analysis was taken from day three of recording at P32 so that animals had substantial time to acclimate to the recording device. We separated the recordings into the light and dark cycles (14:10 cycle), which were analyzed first separately and then together. The data were analyzed over a 24-h period starting at 06:00 AM, when the light cycle begins in the animal facility.

Sleep stages were divided into wake, non-rapid eye movement sleep (NREM), and rapid-eye movement sleep (REM). In addition, NREM and REM stages were combined to study effects on overall sleep versus wake behavior (Sleep). Sleep stages were manually scored in 10 s epochs using Sirenia Sleep Pro (Pinnacle Technology, Inc., Lawrence, KS, United States). Wake stages were characterized by high frequency, mixed amplitude cortical EEG and high frequency signal in EMG when animal was moving (Figure 1B). NREM stages were characterized by low frequency, high amplitude EEG and minimal signal in the EMG (Figure 1C). Finally, REM stages were characterized by high frequency, low amplitude EEG resembling awake periods but with minimal signal in the EMG (Figure 1D). Each epoch was categorized into each stage if >50% of the EEG signal corresponded to that particular sleep stage. Time locked videos were used to confirm behavior.

Sleep architecture was analyzed using various sleep behavioral outcomes. We measured the average sum time spent in each sleep stage as well as the percentage of time spent in each stage. Switching between different sleep stages was measured as the number of transitions between stages. We also measured the number of episodes animals spent in each sleep stage, as well as the mean duration of each episode. A sleep episode was defined as a continuous period of time spent in one stage before transitioning into another different stage. Mean episode length was evaluated by summing the total time spent in each episode and dividing that value by the number of episodes. Sleep scorers were blinded to experimental condition and animal sex.

### Power Analysis

Electroencephalogram waveform data from barrel cortex were scored in 10 s epochs corresponding to sleep scoring. Using Sirenia Sleep Pro software, data underwent 4K fast Fourier transform using a Hann window function to obtain power spectra values. Data were divided into seven frequency bands: delta (1–4 Hz), theta (4–8 Hz), alpha (8–12 Hz), beta (12–30 Hz), low gamma (30–59 Hz), and high gamma (61–100 Hz); 60 Hz was omitted to reduce noise in the EEG signal, since this is the frequency of alternating current in the United States. We also further subdivided beta into two frequency bands: beta 1 (12–20 Hz) and beta 2 (20–30 Hz). Absolute power values were summed for each sleep stage and divided by the number of episodes for that particular stage. To assess changes in relative power between treatment groups, absolute values were normalized to the total power for each sleep stage [Relative Power_beta_ = (Absolute Power_beta_/Total Absolute Power_1__–__100 *Hz*_) × 100]. All absolute and relative power bands were analyzed separately as described by statistical analysis below. However, since we did not find any differences in absolute or relative power in other frequency bands, we focused here only on beta frequency oscillations. In this study we opted for presenting only relative power as we wanted to correct for the large differences in the absolute power between subjects. Absolute and normalized power figures were sorted for outliers using Prism Outlier test set at *P* < 0.01. Statistical outliers were crosschecked to the original EEG to determine if figures were due to normal physiological variation or to EEG artifact. Only outliers that contained artifact were removed from analysis. Three animals met these criteria and were removed from analysis.

### Statistical Analysis

Graphpad Prism 8.0 (GraphPad Software Inc., San Diego, CA, United States) was used for univariate analyses, including assessment of neuroapoptosis, sleep architecture analyses, and power analyses associated with sleep architecture. All data were tested for normality by plotting QQ plots of residuals and testing with Shapiro-Wilk test for normality. Data containing residuals that were not normally distributed were grouped into isoflurane treatment and sham control and analyzed using non-parametric Mann-Whitney Test. Data that had normally distributed residuals were analyzed normally using parametric two-way analysis of variance (ANOVA) for anesthesia treatment and sex (2 × 2 design). Multiple comparisons were further determined by Tukey *Post Hoc* tests. Cohen’s D was computed to assess effect size. All data are presented as means and standard deviations and graphed as means and standard error of mean. Data were considered significant at *P* < 0.05.

## Results

### Acute Neurotoxicity

**FIGURE 2:**
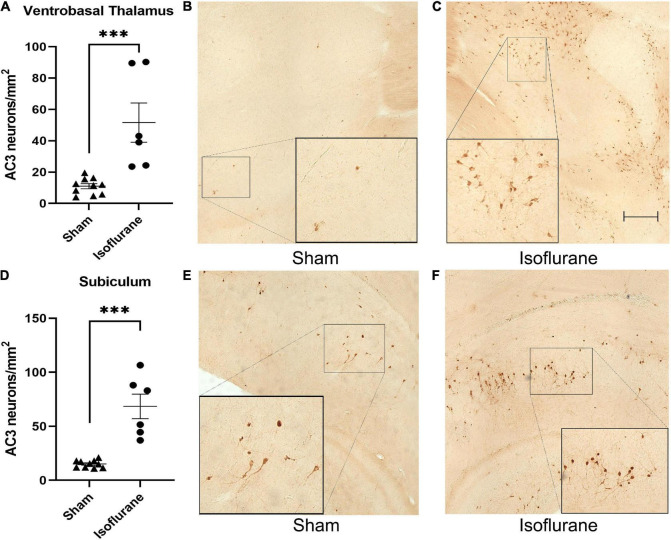
Neonatal isoflurane exposure causes profound acute neurotoxicity. **(A)** Quantification of AC3^+^ neurons showing increased apoptotic profiles in the ventrobasal thalamus of rat pups 2 h after isoflurane exposure (***P = 0.0002). **(B,C)** Side by side representative brain sections of ventrobasal thalamus showing normal physiological apoptosis in Sham animals and high density AC3^+^ neurons in Iso animals. **(D)** Quantification of AC3^+^ neurons showing increased apoptotic profiles in the subiculum 2 h after isoflurane exposure (***P = 0.0002). **(E,F)** Side by side representative brain sections of dorsal subiculum showing basal levels of apoptotic pyramidal neurons in Sham animals compared to neurotoxic levels in Iso animals. Boxed areas represent zoomed in section from original image.

We first determined the short-term neurotoxic effects of a 6-h isoflurane exposure in a cohort of rat pups (Iso *n* = 6, 3 males, 3 females; Sham *n* = 10, 5 males, 5 females). Pups exposed to 1.5% isoflurane for 6 h (51.58 ± 30.66 AC3^+^ neurons/mm^2^) showed a nearly five times increase in neurotoxicity in the ventrobasal thalamus compared to Sham (11.01 ± 5.23 AC3^+^ neurons/mm^2^, Figure 2A), *Mann-Whitney U* = 0, *P* = 0.0002, Cohen’s *D* = 1.844. Few apoptotic neurons are seen in Sham condition (Figure 2B), in which there is normal basal physiological apoptosis occurring. In contrast, we observed more AC3^+^ neurons in the ventrobasal thalamus of Iso treated pups (Figure 2C). This increase was similar in the dorsal subiculum in which Iso pups (68.42 ± 27.90 AC3^+^ neurons/mm^2^) had more apoptotic neurons compared to Sham pups (15.13 ± 3.43 AC3^+^ neurons/mm^2^, Figure 2D), *Mann-Whitney U* = 0, *P* = 0.0002, Cohen’s *D* = 2.681. As with thalamus, there are few AC3^+^ neurons depicted in the subiculum of Sham animals 2 h after anesthesia exposure (Figure 2E), but we observed a large increase in apoptosis in the subiculum of Iso exposed pups (Figure 2F). Because there was no main effect of sex on neuronal apoptosis, data were combined to reflect treatment effect of isoflurane.

### Sleep Architecture

**FIGURE 3:**
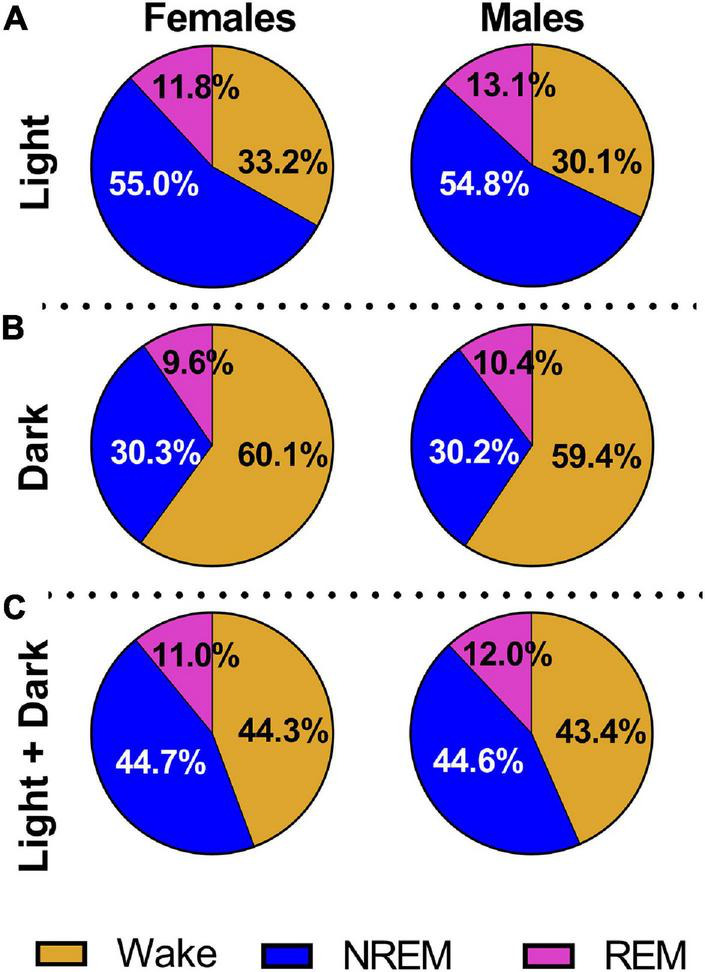
All animals show normal sleep patterns regardless of treatment condition. **(A)** We combined Iso and Sham data to show that males and females displayed characteristic sleep distribution with a greater percentage of time spent asleep during the light cycle. Males also spent a greater percentage of time in REM sleep compared to females (*P* = 0.013). **(B)** Combined Iso and Sham data also show that males and females exhibited normal sleep patterns during the dark cycle, with a greater percentage of time spent active and awake, but no sex differences were noted. **(C)** When light and dark cycles were combined, males spent a higher percentage of time in REM sleep compared to females (*P* = 0.023).

In a second cohort of pups raised to adolescence, we measured the effects of neonatal isoflurane on various sleep architecture measures (Iso *n* = 24, 12 males and 12 females; Sham *n* = 21, 9 males, 12 females). Female and male rats exhibited normal sleep patterns regardless of treatment group. Because rats are nocturnal animals, they rest more during the light cycle and are more active during the dark cycle. This is reflected in our results showing that rats spent a greater percentage of the light cycle sleeping (females = 66.8%, males = 67.9%) and a greater percentage of the dark cycle awake (females = 60.1%, males = 59.4%, Figures 3A,B). However, during the light cycle, males (13.17 ± 0.50%) spent a greater percentage of time in REM sleep compared to females (11.88 ± 0.11%, 2-Way ANOVA, *F*_1_,_41_ = 6.758, *P* = 0.013, Cohen’s *D* = 3.56, Figure 3A). This was not observed in the dark cycle (Figure 3B). When both light and dark cycles were combined, again males spent a greater percentage of time in REM sleep (12.02 ± 0.40%) compared to females (10.93 ± 0.41, Figure 3C, 2-Way ANOVA, *F*_1_,_41_ = 5.580, *P* = 0.023, Cohen’s *D* = 2.69).

**FIGURE 4:**
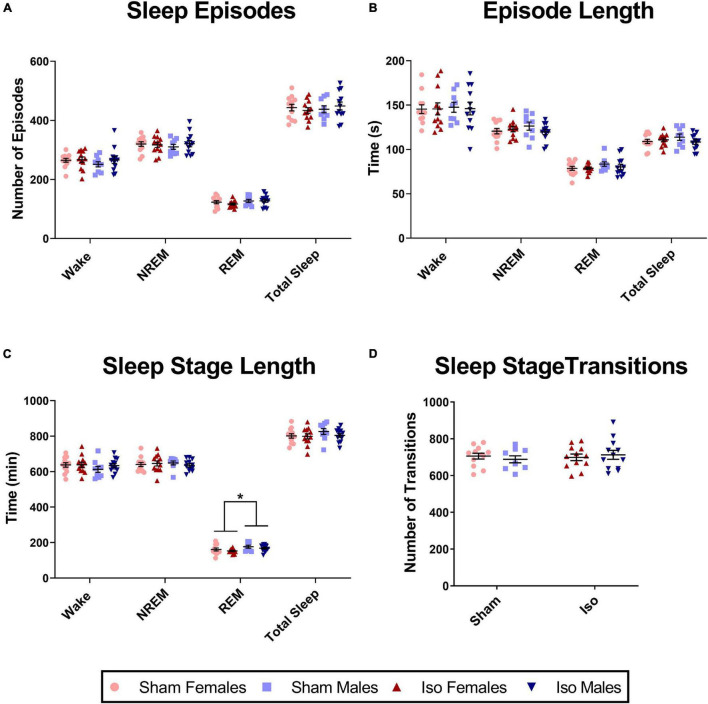
Exposure to neonatal isoflurane does not affect sleep architecture. **(A)** Exposure to neonatal isoflurane did not result in any changes in the number of episodes in the Wake (*P* = 0.374), NREM (*P* = 0.869), REM (*P* = 0.534), and total Sleep stages (*P* = 0.953). **(B)** Corresponding to the number of episodes, the average length of each episode also did not differ between groups for Wake (*P* = 0.917), NREM (*P* = 0.507), REM (*P* = 0.524), and total Sleep (*P* = 0.483). **(C)** Despite sex differences (*P = 0.022), there were no changes in total time spent in each stage after neonatal isoflurane exposure for Wake (*P* = 0.434), NREM (*P* = 0.753), or REM (*P* = 0.210). **(D)** Finally, rats did not differ in transitioning between the different stages, and this was consistent in the light cycle (*P* = 0.658), dark cycle (*P* = 0.820), and when cycles were combined (*P* = 0.659).

Neonatal exposure to isoflurane did not result in any significant changes in sleep architecture across all of the sleep stages; thus, data are presented as combined across cycles. There were no changes in the number of episodes (Figure 4A), the duration of each of those episodes (Figure 4B), the length of individual sleep stages (Figure 4C), or the number of transitions between sleep stages (Figure 4D). However, we did note significant sex differences in the total time spent in REM sleep regardless of treatment condition (2-Way ANOVA, *F*_1_,_41_ = 5.641, *P* = 0.022, Cohen’s *D* = 0.69). Males (172.30 ± 21.68 min) spent a greater amount of time in REM sleep compared to females (157.30 ± 21.85 min, Figure 4C). This was also observed during the light cycle alone (2-Way ANOVA, *F*_1_,_41_ = 6.733, *P* = 0.013, Cohen’s *D* = 0.76), but not in the dark cycle alone (data not shown).

### Power Spectra

**FIGURE 5:**
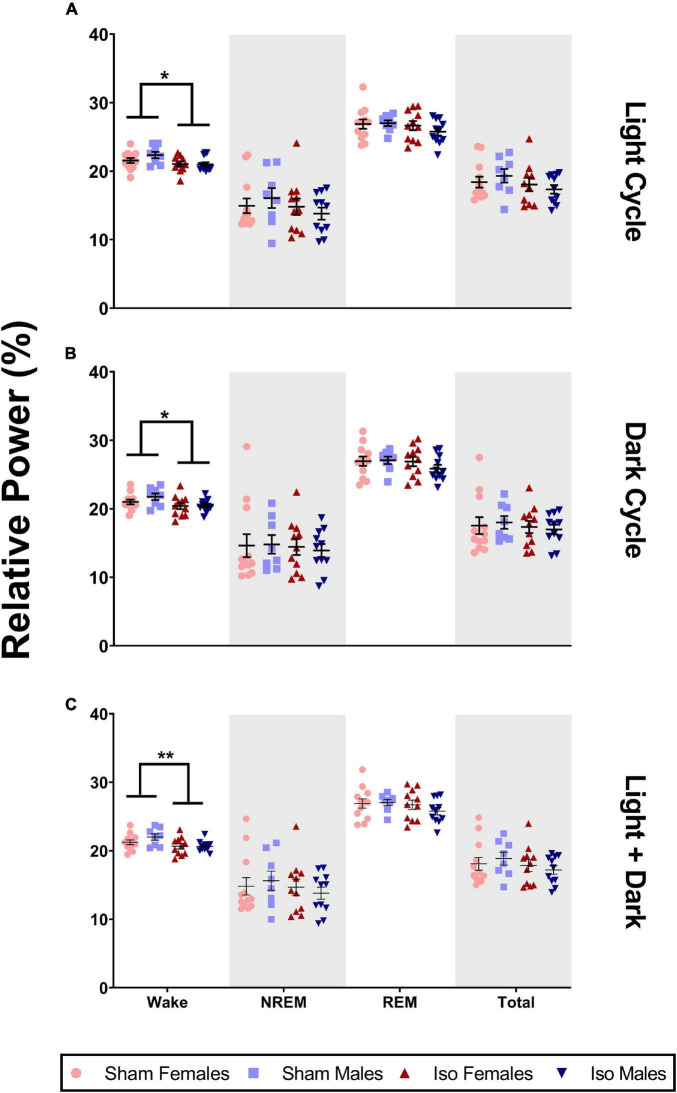
Neonatal isoflurane exposure resulted in a minimal but significant decrease in power spectra in the beta frequency band. **(A)** During the light cycle, there was a decrease in wake beta power in animals exposed to Iso (*P = 0.018). However, there were no changes in beta during NREM (*P* = 0.222), REM (*P* = 0.293), or across total recording period (*P* = 0.188). **(B)** There was also a decrease in wake beta power in Iso exposed rats during the dark cycle (*P = 0.018). But, there were no changes in beta power during NREM (*P* = 0.694), REM (*P* = 0.335), or total recording period (*P* = 0.547). **(C)** Finally, decreases in beta during wake were also observed when both cycles were combined (**P = 0.010), and these changes were not seen during NREM (*P* = 0.420), REM (*P* = 0.252), or total recording period (*P* = 0.276).

Although there were no changes in sleep architecture, we found that exposure to neonatal anesthesia did significantly impact EEG power spectra in the beta frequency range (Iso *n* = 22, 11 males and 11 females; Sham *n* = 20, 8 males and 12 females). Figure 5A shows that during the light cycle, there was a small but significant effect of anesthesia on relative power in the beta frequency band in both male and female rats. Specifically, the animals exposed to isoflurane at P7 (21.02 ± 1.07%) showed lower relative beta power during wake compared to Sham animals (21.875 ± 1.33%, 2-Way ANOVA, *F*_1_,_38_ = 6.158, *P* = 0.018, Cohen’s *D* = 0.71, Figure 5A). These changes were consistent through the dark cycle, (*M*_*Iso*_ = 20.42, *SD*_*Iso*_ = 1.21%, *M*_*Sham*_ = 21.31, *SD*_*Sham*_ = 1.29%, 2-Way ANOVA, *F*_1_,_38_ = 6.071, *P* = 0.018, Cohen’s *D* = 0.71, Figure 5B), and when light and dark cycles were combined (*M*_*Iso*_ = 20.65, *SD*_*Iso*_ = 1.04%, *M*_*Sham*_ = 21.55, *SD*_*Sham*_ = 1.26%, 2-Way ANOVA, *F*_1_,_38_ = 7.391, *P* = 0.010, Cohen’s *D* = 0.78, Figure 5C). We found no statistically significant differences between the Iso and sham cohorts in other bands of EEG power spectra (data not shown).

**FIGURE 6:**
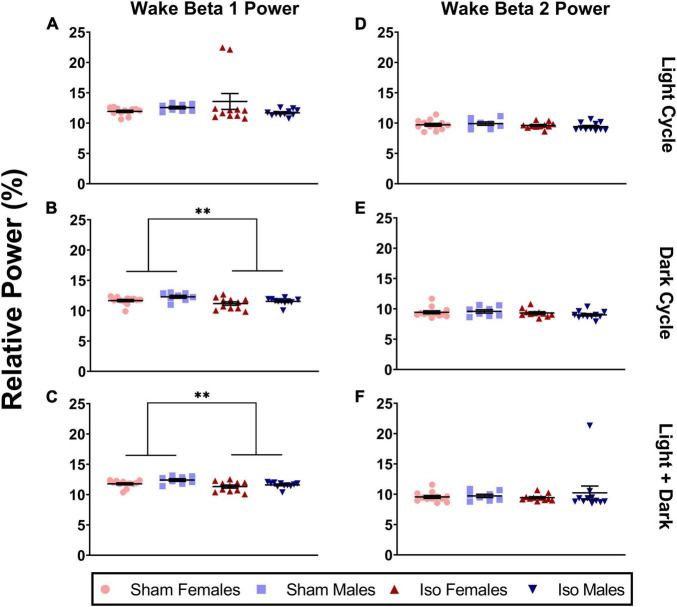
Neonatal isoflurane exposure resulted in a small but significant decrease in power spectra in the beta 1 frequency band, but not in beta 2 during the wake period. **(A)** This was not statistically significant in the light cycle (*P* = 0.076), **(B)** but instead was seen in the dark cycle when animals are most active (*P = 0.001), and **(C)** in both cycles combined (**P = 0.003). For beta 2 oscillations, changes were not statistically significant during **(D)** the light cycle (*P* = 0.173), **(E)** the dark cycle (*P* = 0.215), and **(F)** in both cycles combined (*P* = 0.299).

Because beta oscillations comprise a large range in the EEG, we further subdivided this into two bands, beta 1 and beta 2. Figure 6A shows that during the light cycle, there were no statistically significant changes in beta 1 oscillations (Mann-Whitney, *P* = 0.076). However, during the dark cycle, there was a minimal but significant effect of anesthesia on relative power in the beta 1 frequency band in both male and female rats. Specifically, the animals exposed to isoflurane at P7 (11.35 ± 0.78%) showed lower relative beta 1 power during wake compared to Sham animals (11.91 ± 0.72%, *Mann-Whitney U* = 120, *P* = 0.011, Cohen’s *D* = 0.75, Figure 6B). These changes were also observed when both light and dark cycles were combined (*M_*Iso*_* = 11.45, *SD_*Iso*_* = 0.68%, *M*_*Sham*_ = 12.03, *SD*_*Sham*_ = 0.67%, 2-Way ANOVA, *F*_1_,_38_ = 9.811, *P* = 0.003, Cohen’s *D* = 0.86, Figure 6C). Because the beta frequency range is wide and may have different behavioral correlates, we also analyzed the higher frequency beta 2 separately from the lower frequency beta 1. However, there were no differences between experimental groups in the beta 2 frequency range during the light (2-way ANOVA, *P* = 0.173) or dark (Mann-Whitney, *P* = 0.215) cycle and no changes when both cycles were combined (Mann-Whitney, *P* = 0.299, Figures 6D–F).

## Discussion

We found that exposure to isoflurane during the neonatal period caused profound acute neuroapoptosis, but did not result in changes in sleep architecture during adolescence. Instead, we found small but significant changes in power spectra related to sleep-wake behavior. Specifically, animals exposed to isoflurane as neonates showed lower relative power in the beta frequency band during awake state.

During the first week of rodent life, many synapses are silent, and cortical EEG activity is characterized by quiet periods intermixed with intermittent spindle bursts (Khazipov and Luhmann, 2006). During this period, sleep stages can only be categorized as active and inactive states (Gramsbergen, 1976; Seelke and Blumberg, 2008). During the second week of rodent development, there is an interplay of NMDA and GABA_A_ activity regulating cortical oscillations. First, NMDA and GABA_A_ activity generates and localizes spindle bursts. Second, spindle bursts help in the maturation of key thalamocortical circuits critical in the transition from a discontinuous EEG to mature sleep-wake oscillations (Minlebaev et al., 2007). Traditional anesthetics, such as isoflurane, are GABA_A_ receptor potentiators and can potentially dysregulate this transition. Moreover, during synaptogenesis, neurons rely on a balance of excitatory and inhibitory inputs to strengthen synapses. This period is characterized by differential expression of the sodium-potassium-chloride cotransporter (NKCC1) as well as the potassium-chloride-cotransporter (KCC2), which bring about the switch from excitatory to inhibitory GABAergic transmission (Galanopoulou, 2013; Semple et al., 2013; Oh et al., 2016; Cabrera et al., 2020). In tandem, there is also increased expression of NMDA receptors, and together, these developmental changes regulate neuronal excitability. Introducing pharmacological concentrations of NMDA receptor antagonists and GABA_A_ receptor agonists can disrupt this excitatory/inhibitory balance. Thus, anesthesia-induced neuroapoptosis results in loss of neurons and synapses, including those in thalamus, which are vital to forming and pruning of thalamocortical connections. In this study, we observed profound neurotoxicity after exposure to 6 h of 1.5% isoflurane, consistent with results published by Jevtovic-Todorovic et al. (2003), and we predicted that this could alter sleep architecture.

However, we did not observe any lasting effects of neonatal anesthesia on adolescent sleep-wake architecture. In the current study, both Iso and Sham adolescent rats spent a greater amount of time asleep during the light cycle and a greater amount of time awake during the dark cycle. On average, during the dark cycle, our rats spent slightly less time awake (around 60% of the time) compared to around 67% reported in the literature. Also during the dark cycle, our rats experienced a greater percentage of REM sleep (around 10%) compared to between 3 and 5% reported by others (Frank et al., 2017; Soltani et al., 2019). These differences may be due to differences in the light/dark cycle of our animal facility, which is 14:10 compared to the standard 12:12 light:dark conditions (Frank et al., 2017; Soltani et al., 2019). They may also be due to natural variability due to institutional factors, as others have found the average REM period to consist of about 15% of total sleep during the light cycle and about 10% of total sleep during the light cycle, with variability depending on female estrus cycle (Swift et al., 2020). We also found that males spent more time in REM sleep compared to females, which is consistent with previous work showing that males spend more time in paradoxical sleep, which is analogous to REM sleep (Fang, 2002).

In contrast to our study, Lunardi et al. (2019) found that neonatal rats exposed to midazolam (2 mg/kg), nitrous oxide (75%), and isoflurane (0.75%) for 6 h had an increase in REM sleep during adolescence compared to Sham animals (Lunardi et al., 2019). One key difference between this study and ours may be the use of an NMDA receptor antagonist, nitrous oxide, in addition to the GABA_A_ receptor agonists, isoflurane, and midazolam. The combination of targeting the two major receptor subtypes involved in cortical maturation may have resulted in greater long-term changes than with targeting mostly GABA_A_ receptors with isoflurane in our study. Instead, we found that all our animals exhibited normal sleep patterns. Our current findings with isoflurane are consistent with our previous experiments with exposure to a single NMDA receptor antagonist, ketamine; adolescent rats showed no changes in sleep architecture but exhibited increased gamma oscillations during sleep, further supporting that exposure with a single anesthetic agent may not be sufficient to cause sleep disturbances (Manzella et al., 2020). Another consideration is that our study found sex differences in REM sleep, in which males spent more time in REM sleep compared to females. Although our work and that of Lunardi et al. (2019) contained males and females, it is important to consider properly powering and separating data by sex in order to control for contribution of sex differences to experimental effects.

Although our study strongly suggests that using only isoflurane anesthesia during neurodevelopment does not change sleep architecture, there is still a possibility of circuit dysregulation and changes in oscillatory behavior. Indeed, we observed small changes in the relative beta power during the wake stage of animals exposed to isoflurane. Beta oscillations play an important role in encoding sensory information in rodents and in turn facilitating memory (Fourcaud-Trocmé et al., 2019; Gnaedinger et al., 2019). In primate models, beta burst oscillations in the prefrontal cortex primarily occur during delays between memory tasks and are thought to prevent interference during the learning process (Lundqvist et al., 2018). Further, we divided beta oscillations into beta 1 (12–20 Hz) and beta 2 (20–30 Hz), and we only observed small differences in the lower beta 1 range. These changes appear to be driven by the dark cycle in which the animals are awake longer and more active. Beta 1 oscillations play a role in neuronal physiology underlying short-term memory (Gnaedinger et al., 2019). Beta 1 (around 15 Hz) helps in the formation of neuronal assemblies in the rat association cortex. Specifically, beta 1 oscillations do not require excitatory input to sustain cell assemblies, and instead the oscillations occur during the rebound from inhibition. This allows for preference toward excitation from novel inputs, as more familiar inputs decay more quickly, and these novel inputs further contribute to the beta 1 oscillations and facilitate memory (Kopell et al., 2011). The formation of these cell assemblies and corresponding beta 1 rhythms further contributes to working memory by allowing for buffering of information in parietal cortex between executive inputs and new sensory inputs. Together, these studies suggest that beta 1 plays a role in helping to update and organize sensory information for working memory. We speculate that changes in cortical beta 1 may indicate possible problems with working memory in our animals. However, the changes we observed were small, with a 5% change from baseline beta levels, and it is difficult to infer the biological significance of such changes. Future experiments coupling cortical EEG with working memory behavioral task are needed to clarify this relationship.

In contrast, we did not find any changes in beta 2 oscillations in isoflurane treated animals. These changes are typically associated with object exploration in rodents, which was not tested in our experiments (França et al., 2014). They are also associated with odor learning (Fourcaud-Trocmé et al., 2019). In primates, high beta (beta 2) oscillations can promote synaptic plasticity during both hyperpolarizing and depolarizing phases, which form the foundation for long-term depression and potentiation during learning and memory (Zanos et al., 2018). Interestingly, the model proposed by Kopell et al. (2011) suggests that beta 1 oscillations facilitate formation of cell assemblies independent of synaptic plasticity, indicating different mechanisms between beta 1 and beta 2 oscillatory patterns.

The focus of neuroapoptotic analysis for this study was in the thalamus, which plays a major role in sleep, and in the subiculum, an area particularly sensitive to neuroapoptosis during exposure to neonatal anesthesia. One limitation was that we did not focus directly on cortical sections for quantifying neurotoxicity. However, literature by our group and others consistently find that exposure to isoflurane during the neonatal period causes cortical apoptosis. Seminal work by Jevtovic-Todorovic et al. (2003) shows that rat pups exposed to 6 h of 1.5% isoflurane have profound apoptosis in parietal cortex. Similarly, a 3 h exposure to 1.5% isoflurane at P5 or P7 causes cortical apoptosis in a mouse models (Maloney et al., 2019). These effects are recapitulated in monkey models, which show extensive cortical apoptosis after surgical planes of isoflurane anesthesia during early development (Noguchi et al., 2017; Schenning et al., 2017). Morphologically the dying neurons in both cortex and subiculum can be categorized as primary pyramidal neurons. As mentioned earlier, beta 1 oscillations in the cortex play a role in forming cell assemblies which regulate short term memory (Kopell et al., 2011; Gelastopoulos et al., 2019). We speculate that profound apoptosis of cortical primary pyramidal neurons during development may perturb this circuit and possibly lead to poor learning and memory outcomes later in life.

Indeed, rodents exposed to neonatal anesthesia experience long-term neurocognitive impairment. Our group found that exposure to midazolam, nitrous oxide, and isoflurane resulted in poor spatial memory in both adolescents and adults (Jevtovic-Todorovic et al., 2003). Similarly exposure to the NMDA receptor antagonist, ketamine, results in spatial memory deficits during late adolescence/young adulthood (Atluri et al., 2018). Exposure to neonatal anesthesia also alters non-spatial memory, and rats exposed to propofol as neonates exhibit poor object recognition memory as adolescents (Zhu et al., 2010). Thus, aberrant changes in beta oscillations in our adolescent rats during the awake stage may be indicative of greater circuit dysregulation related to learning and memory. These relationships will be beneficial to explore in future experiments utilizing a combination of EEG or local field potential recordings in tandem with various learning and memory behavioral paradigms.

Another limitation of the current study is that EEG signals were obtained from broader sensory cortex using surface screw electrodes. This can make it difficult to pinpoint the sources of the recorded beta oscillations. Additionally, beta oscillations are not static and can travel and synchronize in other brain regions. In anesthetized animals, beta oscillations in the olfactory bulb and hippocampus are phaselocked and occur simultaneously in a synchronized fashion. These oscillations originate in the olfactory bulb and travel through to the hippocampus through the lateral olfactory tract (Lockmann et al., 2018), indicating the role of beta oscillations in brain connectivity. Beta oscillations are also thought to contribute to brain connectivity between the olfactory bulb and piriform cortex during multimodal learning (Gnaedinger et al., 2019). Hence, it is reasonable to speculate that beta oscillations recorded from barrel cortex in our study could indicate changes occurring in surrounding circuitry. However, future experiments with depth electrodes are needed to explore direct changes in other subcortical brain structures.

In conclusion, our study suggests that exposure to isoflurane during the critical period of synaptogenesis can lead to changes in beta oscillations during the wake state of adolescent rodents. Since beta oscillations play a critical role in cortical development, cognitive processing, and homeostatic sleep regulation we posit that even minimal dysregulation in beta oscillations may be implicated in long-term adverse outcomes associated with neonatal anesthesia.

## Data Availability Statement

The raw data supporting the conclusion of this article will be made available by the authors, without undue reservation.

## Ethics Statement

The animal study was reviewed and approved by Institutional Animal Care and Use Committee at the University of Colorado Anschutz Medical Campus.

## Author Contributions

FM contributed to the conceptualization of experiments, implementation of anesthesia exposures, data analysis, and wrote the manuscript. BG contributed to sleep scoring and data analysis. SM and NU prepared the tissue for AC3 staining, performed the immunohistochemistry experiments, analyzed the corresponding data, and prepared AC3 figure. YR helped to conceptualize and plan sleep experiments and assisted in power spectra analysis. SJ helped to conceptualize the power spectra experiments and assisted in power spectra analysis. VJ-T and ST are the senior authors who played primary roles in conceptualizing the experiments. All authors contributed to the article and approved the submitted version.

## Author Disclaimer

The content is solely the responsibility of the authors and does not necessarily represent the official views of the National Institute of Neurological Disorders and Stroke (NINDS) or the National Institutes of Health (NIH).

## Conflict of Interest

The authors declare that the research was conducted in the absence of any commercial or financial relationships that could be construed as a potential conflict of interest.

## Publisher’s Note

All claims expressed in this article are solely those of the authors and do not necessarily represent those of their affiliated organizations, or those of the publisher, the editors and the reviewers. Any product that may be evaluated in this article, or claim that may be made by its manufacturer, is not guaranteed or endorsed by the publisher.
